# Supplementary material for: Southern-style Pad Thai sauce: From traditional culinary treat to convenience food in retortable pouches
Source: PLoS One. 2020 May 21;15(5):e0233391. doi: 10.1371/journal.pone.0233391 (PMC7241765; doi:10.1371/journal.pone.0233391)
Supplement: S1 Table — (DOCX) [file pone.0233391.s001.docx]

**Table 1. Chemical property of retorted southern-style *Pad Thai* sauce added with different stabilizers**

| Sample | Moisture (%) | a_w_ | pH | PV (meq/kg sample) |
| --- | --- | --- | --- | --- |
| Control | 52.28±1.35^c^ | 0.97±0.00^b^ | 4.97±0.01^c^ | 19.60±0.05^b^ |
| PS + XG | 45.97±0.28^b^ | 0.95±0.00^a^ | 4.95±0.00^b^ | 23.03±5.78^b^ |
| LT | 45.03±1.77^b^ | 0.95±0.00^a^ | 4.38±0.01^a^ | 9.77±0.04^a^ |
| WPI | 40.17±0.15^a^ | 0.95±0.00^a^ | 5.01±0.00^d^ | 19.65±0.33^b^ |

PS + XG =2.3% potato starch + 0.1% xanthan gum, LT = 0.5% lecithin and WPI = 4% whey protein isolate.

Values are given as mean ± standard deviation from triplicate determinations.

Different letters in the same column indicate significant differences (p<0.05).
