# Supplementary material for: Southern-style Pad Thai sauce: From traditional culinary treat to convenience food in retortable pouches
Source: PLoS One. 2020 May 21;15(5):e0233391. doi: 10.1371/journal.pone.0233391 (PMC7241765; doi:10.1371/journal.pone.0233391)
Supplement: S2 Table — (DOCX) [file pone.0233391.s002.docx]

**Table 2. Physical property of retorted southern-style *Pad Thai* sauce added with different stabilizers**

| Sample | Creaming layer (%) | Apparent viscosity (mPa.s) | Color | | | |
| --- | --- | --- | --- | --- | --- | --- |
|  |  |  | *L** | *a** | *b** | Δ*E** |
| Control | 10±1 | 25±1^a^ | 29.52±0.35^a^ | 22.22±0.20^a^ | 38.32±0.85^a^ | 3.88±0.82^a^ |
| PS + XG | ND^#^ | 14167±205^d^ | 31.48±0.07^c^ | 23.14±0.21^b^ | 41.52±0.93^b^ | 5.09±0.80^b^ |
| LT | ND | 2738±18^b^ | 30.60±0.12^b^ | 23.37±0.35^b^ | 39.60±0.48^a^ | 4.48±0.14^ab^ |
| WPI | ND | 5697±35^c^ | 29.33±0.20^a^ | 22.23±0.28^a^ | 38.21±0.99^a^ | 3.91±0.11^a^ |

PS + XG = 2.3% potato starch + 0.1% xanthan gum, LT = 0.5% lecithin and WPI = 4% whey protein isolate.

Values are given as mean ± standard deviation from triplicate determinations.

Different letters in the same column indicate significant differences (p<0.05).

Δ*E** was calculated relative to the color of the original control sauce without retorting. *L**, *a**, and *b** of the original control sauce without retorting were 31.98±0.06, 19.41±0.20, and 38.22±0.54, respectively.

^#^ND, not detected.
