# Supplementary material for: Southern-style Pad Thai sauce: From traditional culinary treat to convenience food in retortable pouches
Source: PLoS One. 2020 May 21;15(5):e0233391. doi: 10.1371/journal.pone.0233391 (PMC7241765; doi:10.1371/journal.pone.0233391)
Supplement: S3 Table — (DOCX) [file pone.0233391.s003.docx]

**Table 3. Sensory property of retorted southern-style *Pad Thai* sauce added with different stabilizers**

| Sample | Color | Viscosity | Odor | Overall liking |
| --- | --- | --- | --- | --- |
| Control | 6.87±1.22^a^ | 4.09±0.64^a^ | 6.47±1.66^a^ | 6.47±1.66^a^ |
| PS + XG | 7.03±0.98^a^ | 6.60±1.07^b^ | 6.77±0.97^a^ | 6.80±1.00^a^ |
| LT | 7.00±0.83^a^ | 6.83±1.18^b^ | 7.00±1.12^a^ | 7.13±1.11^a^ |
| WPI | 7.15±1.03^a^ | 6.70±1.06^b^ | 6.97±1.19^a^ | 7.25±1.08^a^ |

PS + XG =2.3% potato starch + 0.1% xanthan gum, LT = 0.5% lecithin and WPI = 4% whey protein isolate.

Values are given as mean ± standard deviation from 30 determinations.

Different letters in the same column indicate significant differences (p<0.05).
