# Supplementary material for: Southern-style Pad Thai sauce: From traditional culinary treat to convenience food in retortable pouches
Source: PLoS One. 2020 May 21;15(5):e0233391. doi: 10.1371/journal.pone.0233391 (PMC7241765; doi:10.1371/journal.pone.0233391)
Supplement: S4 Table — (DOCX) [file pone.0233391.s004.docx]

**Table 4. Color of retorted southern-style *Pad Thai* sauce added with different antioxidants**

| Sample | *L** | *a** | *b** | ∆*E** |
| --- | --- | --- | --- | --- |
| Control | 29.95 ± 0.15^d^ | 24.66 ± 0.18^b^ | 43.37 ± 0.66^c^ | 7.28±0.22^a^ |
| As + Toc | 28.45 ± 0.31^b^ | 29.28 ± 0.33^d^ | 44.13 ± 0.94^c^ | 11.20±0.24^c^ |
| EDTA | 30.17 ± 0.08^e^ | 30.03 ± 0.95^e^ | 43.92 ± 0.60^c^ | 11.29±0.37^c^ |
| BHT | 29.31 ± 0.27^c^ | 27.82 ± 0.32^c^ | 42.02 ± 1.68^b^ | 8.63±0.62^b^ |
| Mix | 23.67 ± 0.03^a^ | 23.67 ± 0.03^a^ | 35.57 ± 0.13^a^ | 8.51±0.19^b^ |

As + Toc = 500 mg/kg ascorbyl palmitate *+* 500 mg/kg α-tocopherol, EDTA = 100 mg/kg EDTA, BHT = 200 mg/kg BHT, and Mix = 500 mg/kg ascorbyl palmitate + 500 mg/kg α-tocopherol + 100 mg/kg EDTA 200 mg/kg BHT.

Δ*E** was calculated relative to the color of the original control sauce without retorting. *L**, *a**, and *b** of the original control sauce without retorting were 31.67±0.04, 21.01±0.14, and 37.31±0.76, respectively.

Values are given as mean ± standard deviation from triplicate determinations.

Different letters in the same column indicate significant differences (p<0.05).
