# Supplementary figures and images for: Southern-style Pad Thai sauce: From traditional culinary treat to convenience food in retortable pouches
Source: PLoS One. 2020 May 21;15(5):e0233391. doi: 10.1371/journal.pone.0233391 (PMC7241765; doi:10.1371/journal.pone.0233391)

## Slide 1
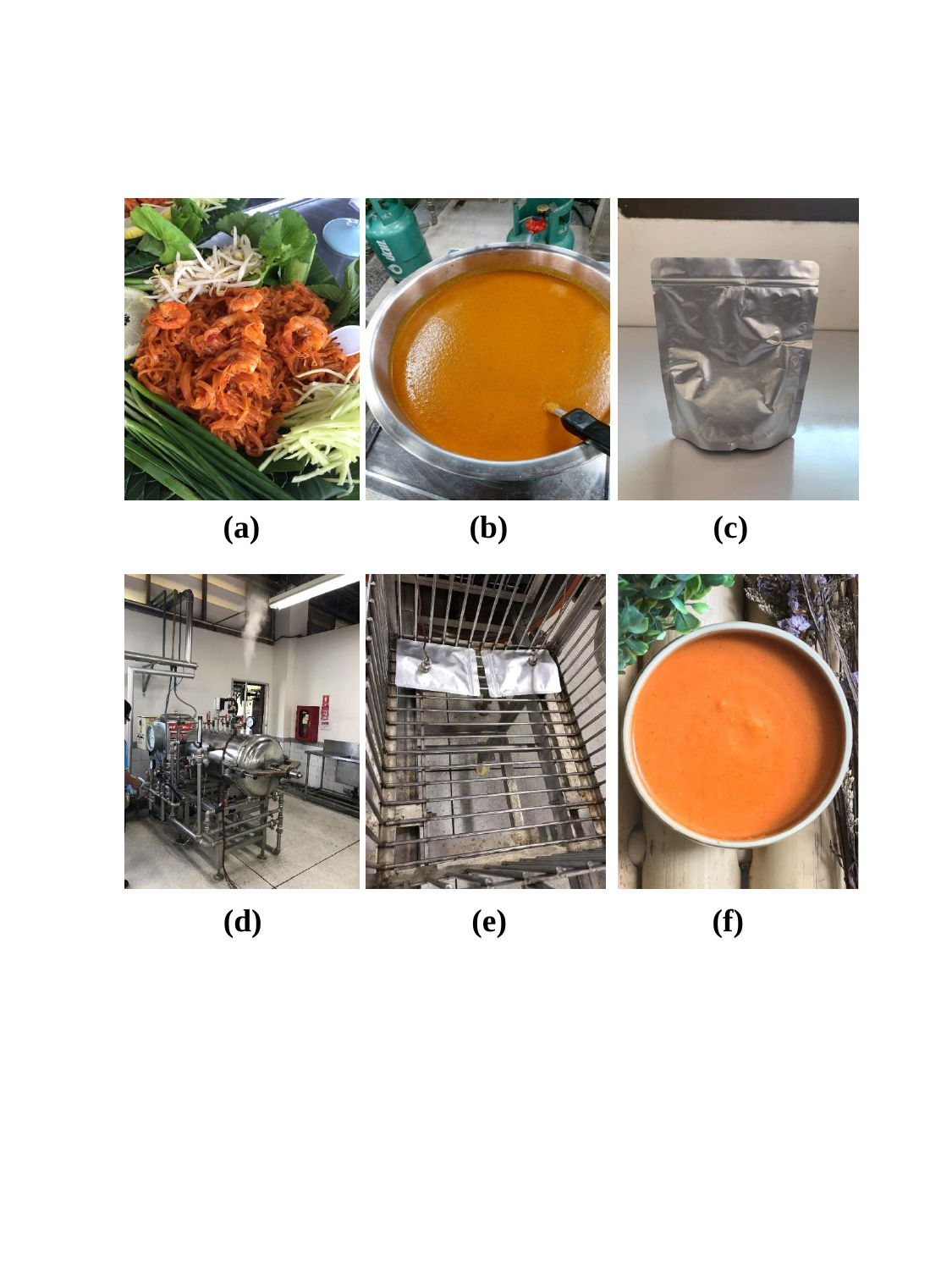

(a)
(b)
(c)
(d)
(f)
(e)

Supplement: S1 Fig — (PPTX) [file pone.0233391.s005.pptx]

## Slide 1
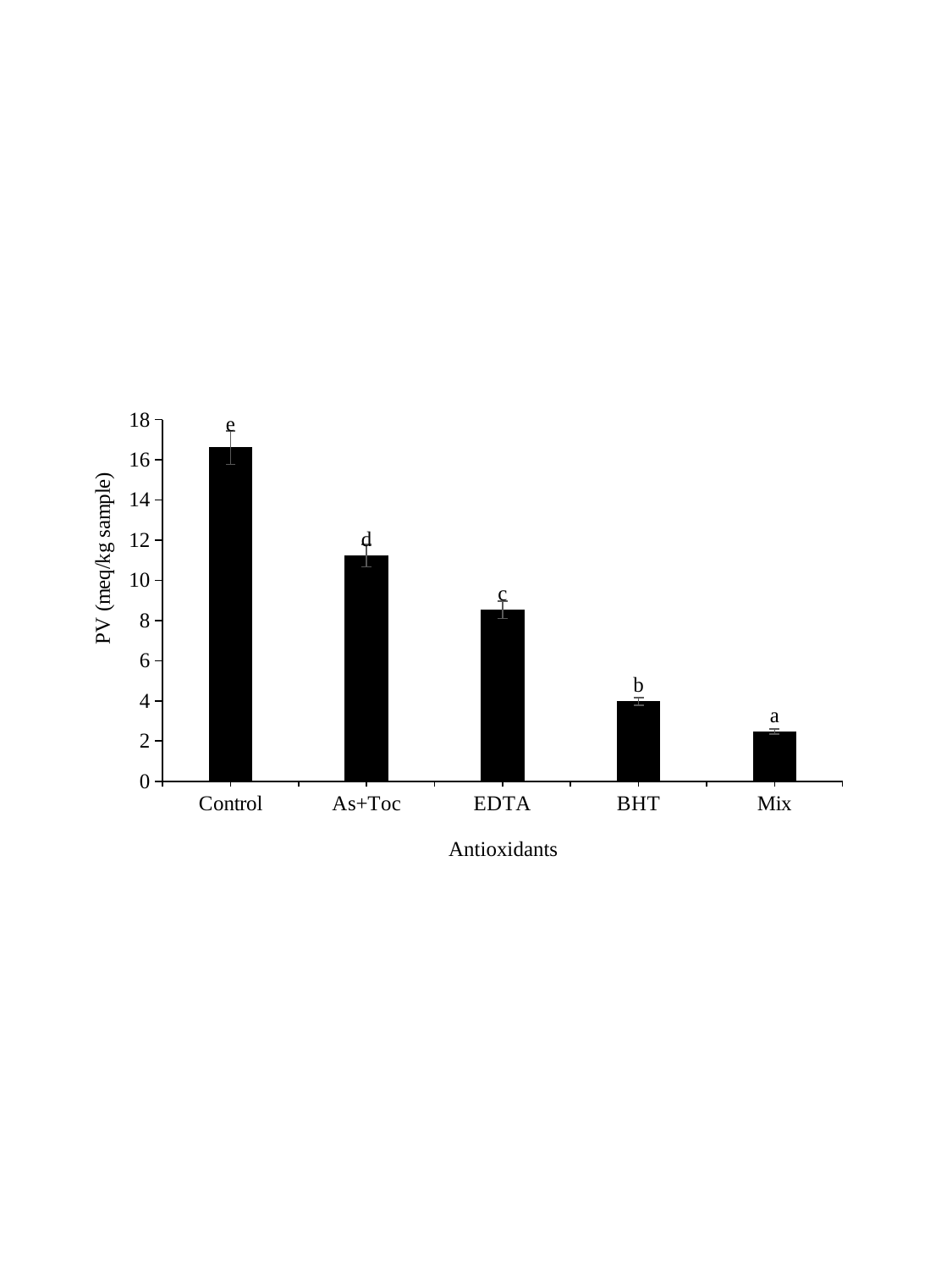

### Chart
| Category | ผ่านการให้ความร้อน |
|---|---|
| Control | 16.6 |
| As+Toc | 11.229999999999999 |
| EDTA | 8.530000000000001 |
| BHT | 3.9699999999999998 |
| Mix | 2.4699999999999998 |Antioxidants

Supplement: S2 Fig — (PPTX) [file pone.0233391.s006.pptx]
